# Spatial- and Temporal-Trajectory Analysis of the Crested Ibis (*Nipponia nippon*) by Fusing Multiple Sources of Data

**DOI:** 10.3390/ani13020237

**Published:** 2023-01-09

**Authors:** Yulong Zhou, Xian Jiang, Zhanlong Chen

**Affiliations:** 1 School of Geography and Information Engineering, China University of Geosciences, Wuhan 430074, China; 2 Institute of Forest Resource Information Techniques, Chinese Academy of Forestry, Beijing 100091, China; 3 School of Computer Science, China University of Geosciences, Wuhan 430074, China; 4 Key Laboratory of Geological Survey and Evaluation of Ministry of Education, China University of Geosciences, Wuhan 430074, China

**Keywords:** behavioral analysis, night roosting points, anniversary activity, trajectory data clustering, trajectory data supplementation, habitat prediction

## Abstract

**Simple Summary:**

This paper provides a methodology for analyzing the trajectory of the Crested Ibis (*Nipponia nippon*): its clustering and mining habitats, trajectory-data supplementation, the analysis of behavioral patterns, and finally, potential habitat mining. It was found that the characteristics of the Crested Ibis habitat in nature reserves include proximity to water sources and cultivated land close to woodlands. Additionally, there is a certain aggregation and sharing of night roosting sites, which are easily disturbed by human activities, and there is a strong correlation between the spatial proximity of the Crested Ibis night. Although human behavior threatens the survival of the Crested Ibis, they still depend on human behavior, mainly for food and water. Moreover, farmers are encouraged to store water in paddy fields in winter and to avoid using harmful substances such as pesticides. Luoshan County provides a protected area for the Crested Ibis; it was analyzed and found to be appropriately located, with an excellent overall habitat-rating. The Chinese Government has also set up corresponding ecological and environmental protection zones, and it is essential to provide solutions for the reintroduction of the Crested Ibis.

**Abstract:**

The Crested Ibis (*Nipponia nippon*) is an endangered animal with an extremely high ecological, humanistic, and scientific value. However, this species still faces survival challenges, due to rapidly shrinking foraging grounds, the serious interference of human behavior, and increased habitat requirements. Geographical environment is a significant factor affecting Crested Ibis behavior-pattern analysis and habitat protection. The spatial and temporal trajectory contains habitat location and period information, a vital record of the Crested Ibis’ habits, and the basis of all research. Nevertheless, there are only a handful of studies on the missing trajectory data and fusing multiple sources of environmental data-research methods. We studied the spatial and temporal habitat use of the tracked Crested lbis by fusing multiple data-sources in China. This paper adopts the LSTM (long short-term memory) model to supplement the missing trajectory data and perform cluster mining; and a random forest model is used to predict the habitat of the Crested Ibis with high fitting accuracy (*R*^2^ = 84.9%). The results show that the Crested Ibis distribution-pattern is characterized by high altitude and proximity to woodland and rivers. Additionally, the habitat dependence on the village implicates human agricultural activities in positively impacting its reproduction. This paper provides a complete method for analyzing Crested Ibis’ spatial and temporal trajectory by fusing multi-source data, which is crucial for protecting the survival and reproduction of the Crested Ibis.

## 1. Introduction

Whilst not critically endangered, many animals are facing a very high probability of extinction in the wild in the near future [1]. The protection of endangered species constitutes an important part of the ecosystem, and the first task in this regard is habitat protection and restoration [2].

The Crested Ibis (*Nipponia nippon*), a medium-sized wading bird that was once distributed in East Asia, across China, Japan, Russia, Korea, and South Korea, is one of the most endangered birds in the world, and is classified as a Class I national priority animal [3]. In 2019, it was registered as a vulnerable species (VU) on China’s Red List and as endangered by the International Union for Conservation of Nature (IUCN) [4], due to its extremely high ecological, humanistic, and scientific value [5]. Its population has been on the brink of extinction for approximately a century [6]. However, by 2021, its numbers reached more than 7000 [7]. This is mainly due to the increased efforts to protect the natural environment of the Crested Ibis.

Understanding environmental influence is practical for the reproduction of the Crested Ibis. High-quality habitats can improve the survival ability and ensure the reproduction of Crested Ibis populations [8]. Altitude and wetlands greatly impact the Crested Ibis’ survival [6,9]. Changes in vegetation cover will affect the distribution and reproduction of the Crested Ibis [10]. Human activities should also be considered [11]. The Crested Ibis relies greatly on local farmers [12] and tends to retain its previous nests [13]. Therefore, farmland is vital to maintaining their populations [14,15], as they prefer to forage in shallow rivers [16,17], where food resources have recovered due to a ban on pesticide use [7]. Therefore, based on the spatial and temporal trajectories of the Crested Ibis, it is vital to explore and evaluate their habitat environment to ensure a sufficient and suitable habitat during the conservation and migration process [18]. 

Some implicit behavioral and activity patterns, potential information on habitat distribution and selection, population dynamics, and group behavior can be discovered through the spatial and temporal trajectory-data analysis of the Crested Ibis [19,20]. Research methods such as comprehensive habitat evaluation [3], maximum pixel method [10], automatic identification method [21] and the Maxent model [22,23], in which Wang Qi et al. [10] explored the vegetation characteristics of Crested Ibis habitats. A manipulative experimental method was adopted to summarize the minimum approach area for tourists, to discover the Crested Ibis’ tolerance to human behavioral disturbance during their wandering period [24]. The Crested Ibis has a certain adaptability and some requirements for living conditions in the wild. LU Shaohui et al. found the breeding factors of the Crested Ibis in the Dongzhai Nature Reserve in Henan, including weather, altitude and other interference factors [25]; Hu et al. evaluated and estimated potential collisions on highways and railways by observing the behavioral trajectories of the Crested Ibis [26]. There are also some studies that discuss the survival status of the Crested Ibis after release, and, based on Crested Ibis activity data, explore the best release strategies for the Crested Ibis [27], reproductive differences in the reintroduction of the Crested Ibis [28], and the Crested Ibis population viability [29]. 

However, some potential problems need to be addressed. Firstly, the problem of the missing trajectory of the Crested Ibis has not been effectively resolved. Moreover, multiple environmental data sources should be integrated, which can help understand the interactions between individuals and their environment, providing decisions and a basis for the conservation and management of the Crested Ibis. Finally, the Maxent model, developed by Steven J. Phillips, does not include Asia in its training data [30]. Additionally, the Crested Ibis suitability for species endemic to Asia remains to be proven, with overfitting problems and a low transferability [31]. Therefore, supplementing missing data, fusing multi-source environmental data, and finding suitable models to predict potential habitats, are required.

This paper integrates the spatial and temporal trajectory-data of the Crested Ibis with multiple environmental data sources. The study was conducted in Luoshan County in Henan Province, Yang County, Hanzhong City, and Chenggu County in Shaanxi Province in China. To address the problem of uneven data distribution caused by the missing trajectory data, an LSTM (long short-term memory) model was built, based on latitude and longitude location information to supplement missing data and ensure its integrity. The biological patterns of the Crested Ibis were analyzed, and a random forest model suitable for the Crested Ibis was designed and implemented, to predict the potential habitat distribution of the Crested Ibis in Luoshan County, Henan Province, China. Our paper can provide a complete method for an analysis of the trajectory of the Crested Ibis and suggestions for protecting the survival and reproduction of the Crested Ibis.

## 2. Materials and Methods

**Figure 1:**
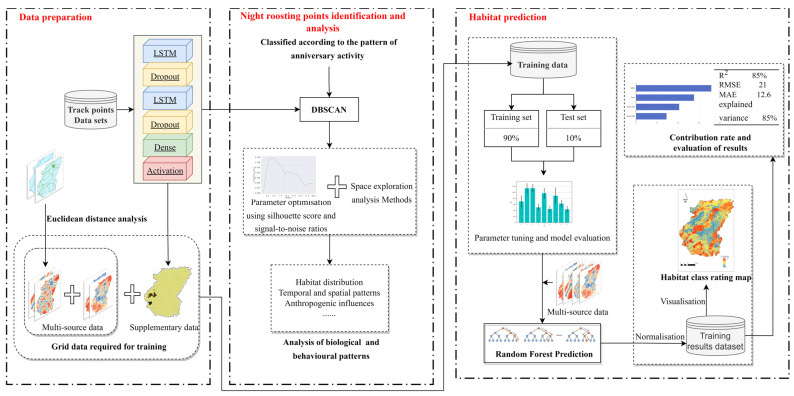
The main methodological framework of the study.

The main methodological framework of the study is shown in Figure 1. Firstly, the spatial and temporal trajectory-data of the Crested Ibis were supplemented by the LSTM (long short-term memory) model. Secondly, according to the anniversary activity, the parameter-tuned DBSCAN (density-based spatial clustering of applications with noise) method was combined with the spatial exploration analysis to obtain the results of the analysis of biological and behavioral patterns of the Crested Ibis. Finally, a random forest model was trained to predict the Crested Ibis habitat. The habitat prediction results were normalized into ranks 1~10 for evaluation, and the final habitat-rank evaluation map and the contribution rate of impact factors were calculated to analyze the future potential habitat-distribution-range of the Crested Ibis.

### 2.1. Study Area and Data

**Figure 2:**
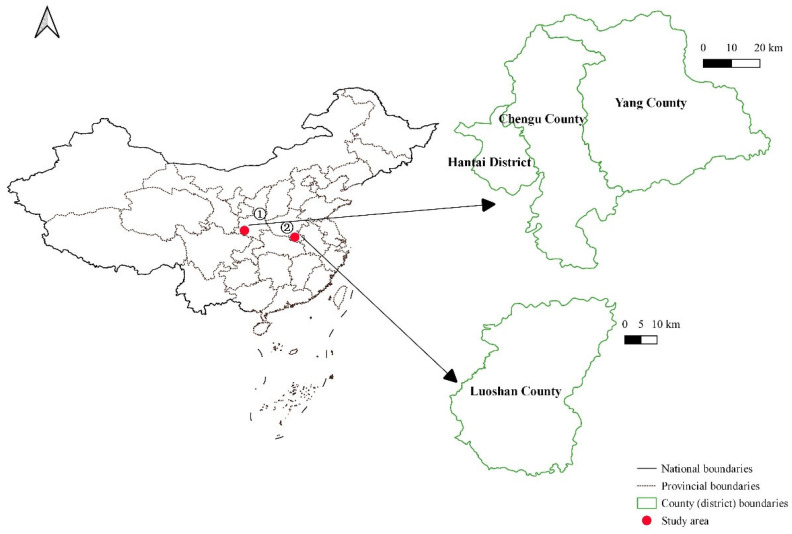
Location of the study area; ① in Shaanxi Province and ② in Henan Province.

We used BeiDou/GPS-enabled tracking backpacks (HQBG3621L) to collect spatial and temporal trajectory-data. The backpacks can automatically transmit the longitude, latitude, and flight times of the Crested Ibis once per hour. In this study, we collected the trajectory data of 7 individuals of the Crested Ibis, a total of 31,825 trajectory data (Table 1), from July 2014 to December 2021, and the study area included Luoshan County in Henan Province, Yang County, Hanzhong City, and Chenggu County in Shaanxi Province (Figure 2). The Dongzhai National Nature Reserve and Hanzhong National Nature Reserve were also in the study area.

Multi-source environmental data were collected to study the interaction between the Crested Ibis and its environment and to analyze its biological patterns and behavior (Table 2). They were DEM (digital elevation model) data, NDVI (normalized difference vegetation index) vegetation-index data, OSM (open-street map) road data, river-distribution vector data, and GlobeLand30 land-cover data (Table 2). The data set of DEM in Geospatial Data Cloud was ASTER GDEM 30M and the location was Luoshan County, Xinyang City, Henan Province. The data set of NDVI in Geospatial Data Cloud was MOD13Q1 250M and the location was Luoshan County, Xinyang City, Henan Province. The download map sheet in GlobeLand30 was N48_25, N48_30, N48_35, N49_25, N49_30, N49_35, N50_25, N50_30, and N50_35. The location of the vector data was Luoshan County, Xinyang City, Henan Province.

### 2.2. Trajectory Data Supplement

An LSTM model was constructed to supplement missing trajectories, to complete the data preparation work (Figure 1, left). Clearing the failed trajectory data and the different data sampling-time intervals resulted in missing trajectory data. Therefore, the LSTM complementary-data model was constructed. The latitude and longitude sequence-data was input. In this study, a six-layer sequence network model was built, with only adjacency between layers and no cross-layer connections, consisting of two layers of LSTM and two layers of dropout, and finally, an added dense layer and an activation layer. The input sequence data were entered into the LSTM for calculation in the first step, and the neurons were randomly removed by the dropout layer with a probability of 0.3 in the second step. After repeating the above two steps, the fully connected layer and activation layer output the final predicted latitude and longitude sequences.

The LSTM model adopted the loss function of MSE (mean squared error) (Supplementary Figure S1).

(1) $$MSE=\frac{1}{m}\overset{m}{\underset{i=1}{\sum }}{\left(y^{\left(i\right)}-\hat{y^{\left(i\right)}}\right)}^{2}$$

The distance error of the test set also needed to be evaluated, using the haversine method [36], where $\hat{lat}$ is the predicted latitude, *lat* is the true latitude, $\hat{lon}$ is the predicted longitude, and *lon* is the true longitude:

(2) $$D=2r\arcsin\left(\sqrt{\sin^{2}\left(\frac{\hat{lat}-lat}{2}\right)+\cos(\hat{lat})\cos\left(lat\right)\sin^{2}\left(\frac{\hat{lon}-lon}{2}\right)}\right)$$

### 2.3. Night-Roosting-Points Identification and Analysis

After the data supplement was completed, it was necessary to identify and analyze the night roosting points of the Crested Ibis (Figure 1, middle). This paper classified the spatial and temporal trajectory-data of the Crested Ibis by period. According to the definitions in the International Crested Ibis Conservation Workshop [5], location points were categorized as night roost points, foraging points, and outing points, according to different periods in different months. The distance between adjacent points greater than 1 km represented different night roosting/foraging/outing points (Table 3) [37]. In this paper, the annual activity of the Crested Ibis was divided into wintering, reproductive and colonial periods. The reproductive period lasts from February to June, the colonial period from July to October, and the wintering period from November to January. Finally, the DBSCAN (density-based spatial-clustering-of-applications-with-noise) clustering algorithm was adopted for use on the Crested Ibis data.

Compared to the K-means method, the DBSCAN [38] method has good adaptability to non-convex clusters, its clustering benefits are unaffected by noise, and the clustering results achieve global optimality [39,40]. The DBSCAN algorithm selects night roosting points and marks noise (Supplementary Figure S2, left). Therefore, compared to the K-means method (Supplementary Figure S2), the clustering algorithm of DBSCAN, which is suitable for the spatial and temporal trajectory-data of the Crested Ibis, was used to identify its habitat and noise points that may be disturbed by human factors, after tuning it according to the characteristics of the Crested Ibis. To evaluate the suitability of the clustering parameters for the Crested Ibis’ spatial and temporal data, the silhouette coefficient proposed by Peter J. Rousseeuw (1986) was used [41]. The closer the silhouette coefficient is to 1 and the noise ratio is smaller, the better the suitability of the clustering parameters for the spatial and temporal data of the Crested Ibis (Supplementary Figure S3). Based on the algorithm’s results, the behavioral patterns and biological patterns of the Crested Ibis were initially analyzed by fusing multiple environmental-data sources.

### 2.4. Habitat Prediction Based on Random Forest

Finally, the trajectory data were further used for habitat prediction to study and evaluate the habitat status (Figure 1, right). We researched in Luoshan County only. Random forest is an integrated algorithm [42] based on bootstrapping in statistics, evolving the bagging method, which combines CART (the Classification and Regression Tree) and random subspace methods to construct a specific number of unrelated trees. The random forest method can prevent the overfitting problem caused by too much information and too high a correlation between trees. It achieves this by combining multiple decision trees and voting or taking the mean value of the decisions’ results to obtain the model’s final result.

The input training-set sample *D_sample_* = {*X*_1_, *X*_2_, *X*_3_, *X*_4_, *y*}; *X*_1_ is the DEM data, *X*_2_ is the Euclidean-distance measure for roads, *X*_3_ is the Euclidean-distance measure for rivers, *X*_4_ is the vegetation index, and *y* is the number of Crested Ibis points in each grid. Training samples were obtained by repeated multiple sampling with put-back using the bagging method, and this sampling result was used as the training set to generate the decision tree. Using the four input feature-variables, *n* feature variables (*n* < 4) were randomly selected for each node in the tree. The best splitting point for the decision tree was determined with the selected *n* feature-variables. Each decision tree (classifier) was grown to its maximum capacity but not pruned, and the results of all decision trees were finally averaged, to construct a regression model.

The metric used to generate CART trees for this study is MSE [42], which determines the divided features, where *D* is the sample dataset, *a* is the input features, *T_a_* is the mean set of the decision tree, $y_{t}^{v}$ is the subset labels, and $\hat{y_{t}^{v}}$ is the mean value of the subset labels:

(3) $$MSE\left(D,a\right)=\underset{t\in T_{a}}{\min}\underset{v\in \left\{-,+\right\}}{\sum }{\left(y_{t}^{v}-\hat{y_{t}^{v}}\right)}^{2}$$

The smaller the value obtained above, the better the sample-set in the tree fit to find the optimal partitioning attribute.

(4) $$a_{best}=argmin_{a}MSE\left(D,a\right)$$

The optimal parameters to construct the model were selected (Supplementary Figure S4, Tables S1 and S2). Additionally, the evaluation metrics of the stochastic forest-habitat prediction model, *R*^2^, *RMSE*, *MAE*, and explained variance were chosen. The mean squared error *RMSE* (root-mean-square error) and *MAE* (mean absolute error) were adopted, to measure the difference between predicted and true values. For the random-forest-regression method, it is not sufficient to only explore the accuracy of the data prediction, but it is also necessary to observe whether the model learns the distribution and pattern of the data, and thus the explained variance and *R*^2^ are needed to evaluate the fitting ability of the model.

(5) $$R^{2}=1-\frac{\sum_{i=1}^{n}{\left(\hat{y^{\left(i\right)}}-y^{\left(i\right)}\right)}^{2}}{\sum_{i=1}^{n}{\left(\bar{y}-y^{\left(i\right)}\right)}^{2}}$$

(6) $$MAE=\frac{1}{n}\overset{n}{\underset{i=1}{\sum }}\left|y^{\left(i\right)}-\hat{y^{\left(i\right)}}\right|$$

(7) $$RMSE=\sqrt{\frac{1}{n}\overset{n}{\underset{i=1}{\sum }}{\left(y^{\left(i\right)}-\hat{y^{\left(i\right)}}\right)}^{2}}$$

(8) $$explained\_variance=1-\frac{Var\left\{y^{\left(i\right)}-\hat{y^{\left(i\right)}}\right\}}{Var\left\{y^{\left(i\right)}\right\}}$$

*Var* denotes the variance of the model.

## 3. Results

### 3.1. Trajectory-Data Supplementation

**Figure 3:**
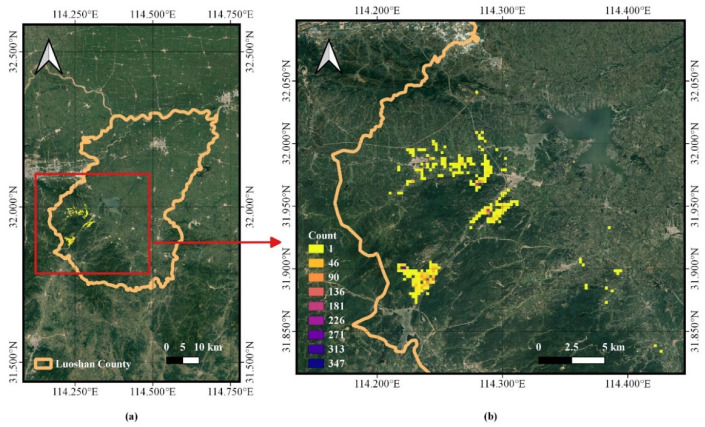
Trajectory supplementation diagram. The red rectangle in (**a**) is enlarged to (**b**). The value of each pixel unit in this map is the number of trajectory points with a resolution of 200 m; the higher the number, the darker the color.

After supplementation, the Crested Ibis trajectory data were mainly located in the central and western regions of Luoshan County (Figure 3).

**Figure 4:**
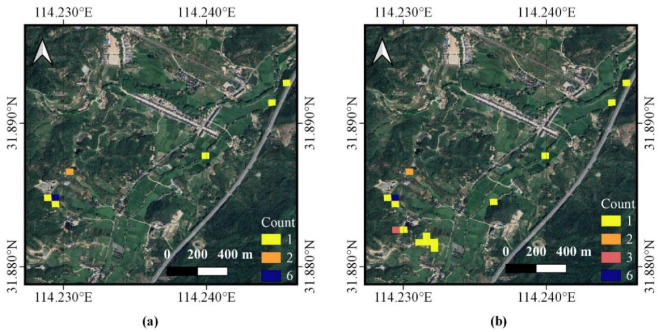
Trajectory supplementation diagram (detail). The picture shows the trajectory data for one day: (**a**) denotes the missing data, (**b**) denotes the supplemented data. The value of each pixel unit is the number of trajectory points.

With further study for additional details, we were able to find missing parts of the trajectory data (Figure 4a) and the supplementation of the data (Figure 4b). In the end, the trajectory data of the Crested Ibis were well supplemented.

### 3.2. Spatial Patterns of Trajectory Points

**Figure 5:**
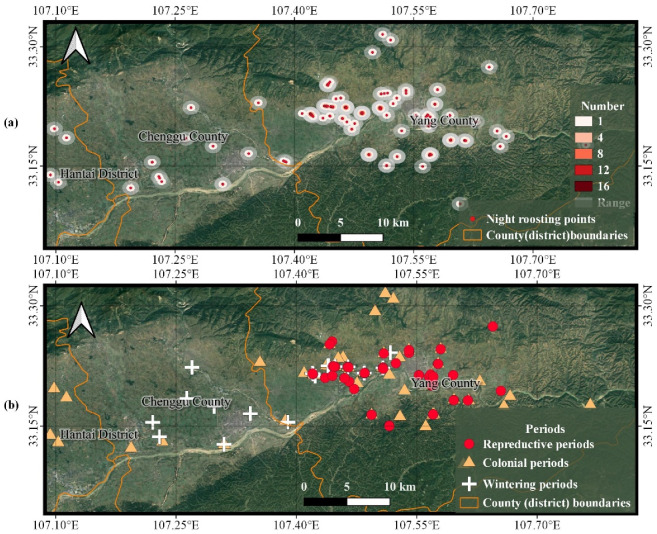
(**a**) Night roosting points in Hantai District, Chenggu County, and Yang County. (**b**) Distribution of activity periods at night roosting points. (**a**) The gray areas indicate the extent of their night roosting points. The darker the red, the greater the number of night roosting points. (**b**) The reproductive period lasts from February to June, the colonial period from July to October, and the wintering period from November to January.

The trajectory data were clustered and noise was removed by DBSCAN for further analysis. The distribution of night roosting points for the Crested Ibis in Hantai District, Chenggu County, and Yang County tended to follow the rivers, with cultivated fields and ponds close to the rivers (Figure 5a). There were also a few more dispersed night roosting points in the north and south, and as they were in their wandering periods, they tended to select a wider range of night roosting points, with a tendency to choose areas at higher altitudes with taller and bushier trees.

The overall night-roosting-points range of the Crested Ibis was dense, with areas of night roosting points generally characterized by proximity to paddy fields, ponds, and surrounding woodland, providing both night roosting points and foraging for the Crested Ibis (Figure 5b). During the reproductive period, the choice of night roosting points fluctuated, with the birds roaming to more densely wooded areas.

The rest of the night roosting points in the three activity periods were similar in distribution, with Hantai District, Chenggu County, and Yang County in Shaanxi Province as the representative areas. Among the three activity periods of the Crested Ibis, the colonial period existed in the widest range, with a tendency to spread to higher altitudes on the north and south sides (Figure 5b). The reproductive period was more concentrated in the central parts of Hantai District, Chenggu County, and Yang County and was closer to the rivers than the colonial and wintering periods (Figure 5b). Additionally, there was a strong correlation between the spatial proximity of the Crested Ibis night roosting points and activity periods.

**Figure 6:**
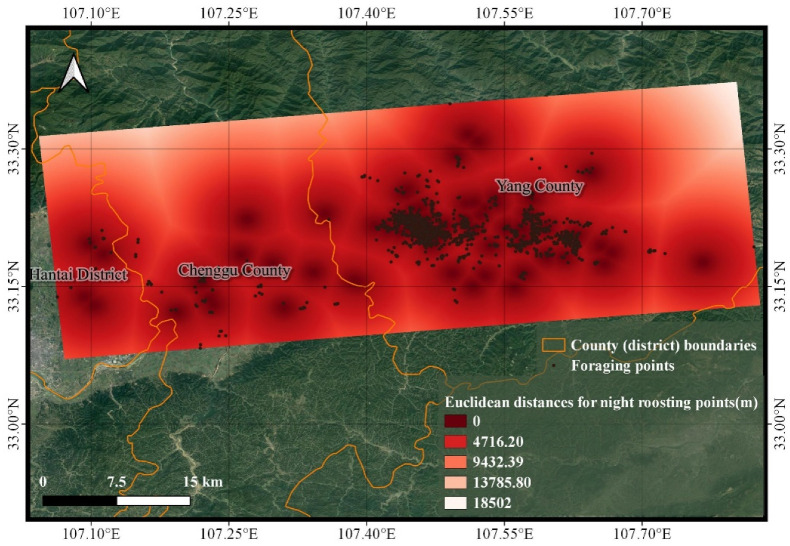
Analysis of the distance between the night roosting points and the foraging points. The Euclidean-distance analysis of the night roosting points in this region shows that the darker the color of the foraging points, the closer the foraging point is to the night roosting points.

Taking Hantai District, Chenggu County, and Yang County in Shaanxi Province as representative regions, the distance-analysis pattern between the night roosting and foraging points was similar for the remaining two locations (Figure 6). Additionally, the foraging points that were very close together were selected very close together, and concentrated around the night roosting points.

Further studies showed a concentration of distances between 60 m and 595.5 m selected for the two kinds of points, with an interquartile spacing of 535.5 m and even a zero distance between them, indicating that the two kinds of points almost overlapped. The standard deviation of 555.9 m indicated a concentrated distance relationship between them and a weak dispersion of data. The mean value of 411 m further indicated the proximity of the night roosting points to the foraging points. With a maximum distance of 5796 m, this represented only 0.1% of the total data, and was most likely a result of the Crested Ibis finding other foraging points to feed at.

### 3.3. Potential Habitat Mining

The accuracy evaluation indicators of the random forest model: The *R*^2^ was 84.9%, and the explained variance was 85.7% (Table 4), indicating that the random forest model designed in this experiment performed well in fitting the data from the Crested Ibis. The *RMSE* was 21.0, and the *MAE* was 12.6 (Table 4), indicating that the difference between the predicted and true values was small, and the prediction model was relatively accurate.

**Figure 7:**
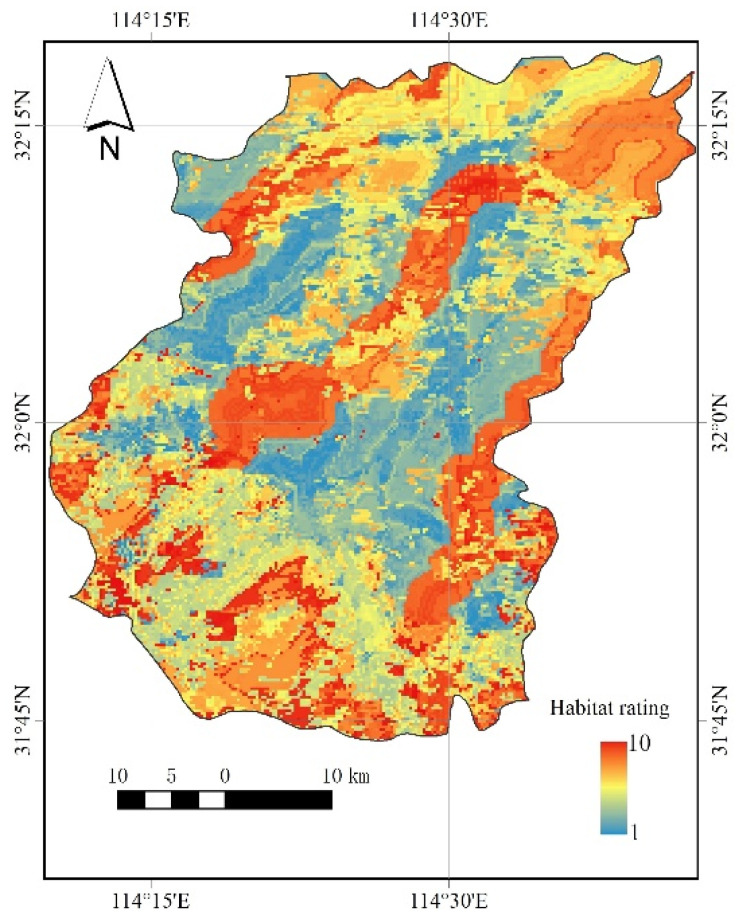
Crested Ibis habitat-rating map. The red indicates the most suitable places for a high habitat-rating; the blue indicates the least suitable places, for a low habitat-rating.

In the southern part of Luoshan County, areas with high DEM (digital elevation model) and high vegetation cover provide foraging grounds for the Crested Ibis. In the southern part of the county, there is a certain amount of woodland cover, which is also more suitable for the Crested Ibis to roost and breed (Figure 7). In Luoshan County, the anthropogenic impact on the Crested Ibis is both positive and negative, as the Crested Ibis does not like to be disturbed by humans, so the habitat level of the Crested Ibis is lower in areas closer to roads but slightly higher in areas closer to rivers, where artificial paddy fields and ponds provide foraging grounds for the Crested Ibis.

In addition to the vegetation cover and elevation data, the location of the habitat with respect to roads and rivers should also be considered when selecting habitats for the Crested Ibis. When selecting future conservation sites for this species, one may prefer areas with a higher vegetation-cover and elevation that are located a certain distance from rivers and roads, to ensure a better foraging area for the Crested Ibis while providing a night roosting environment.

**Figure 8:**
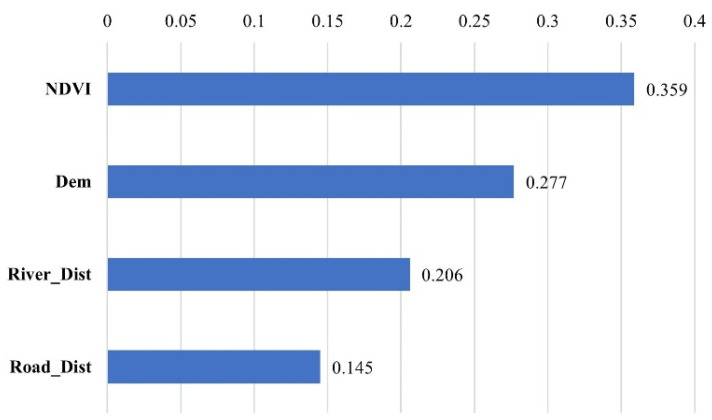
Impact factor contributions. The vertical coordinates River_Dist and Road_Dist indicate the Euclidean-distance assessment for roads and rivers.

Further quantitative analyses of each of the drivers of Crested Ibis habitat-class evaluation were carried out, with larger values indicating a greater influence of this variable on Crested Ibis habitats (Figure 8). The most influential variable in predicting Crested Ibis habitats based on the random forest model is the vegetation index NDVI (normalized difference vegetation index) (0.359), which reflects the degree of variation in the quality of the region’s ecological environment. Additionally, the NDVI has the greatest influence on the selection of Crested Ibis habitat, indicating a strong correlation between Crested Ibis habitat and the degree of vegetation cover. Next is DEM (0.277), which showed that the Crested Ibis is better adapted to higher altitudes and prefers higher-altitude areas for night roosting points. The remaining two variables are River_Dist (0.206), an assessment of Euclidean-distance to rivers, and Road_Dist (0.145), an assessment of Euclidean-distance to roads (Figure 8). Areas closer to rivers and roads, where there is more water and arable land, often meet the dietary needs of the Crested Ibis.

**Figure 9:**
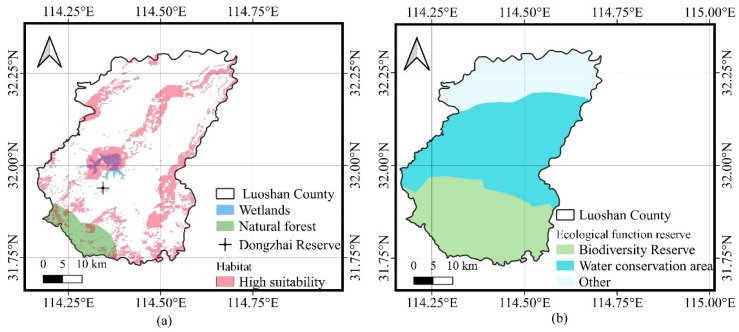
(**a**) High-suitability habitat; (**b**) Ecological function reserve.

We also found that a very large part of the wetlands is a highly suitable habitat. In addition, the southern part of Luoshan County, where the natural forest is located, corresponds to a wider range of habitats, with a high suitability in the northern part, and the southern part is more suitable for a Crested Ibis habitat than the northern part. At the same time, we also found that Dongzhai Reserve is located between wetlands and natural forests in Luoshan County (Figure 9a). It is a very advantageous geographical location, and an excellent place for the Crested Ibis to inhabit.

The extensive coverage of the ecological function of reserves protects most habitats of high suitability. The Dongzhai Reserve is in the southern part of Luoshan County and is covered by a biodiversity reserve, and a water conservation area also covers the area where the wetlands are located (Figure 9b). The water conservation area in Luoshan County will not only protect the local production and living environment, but also better guarantee the habitat suitability for the survival of the Crested Ibis. It shows that Luoshan County is relatively good in terms of environmental protection, ensuring the survival of the Crested Ibis.

## 4. Discussion

We collected a large number of the Crested Ibis trajectory data in the Henan and Shaanxi provinces of China from 2014 to 2021 with the tracking backpacks. By analyzing the data, we find that the Crested Ibis prefer cultivated lands close to forest land, paddy fields, and ponds, for the night. This is associated with the food provided by paddy fields and ponds for the Crested Ibis [6,7,9,12,15]. The areas with a high concentration of foraging points can provide more night roosting points, so the night roosting points of the Crested Ibis are close to areas with a high concentration of paddy fields and ponds. Another finding is that the activity areas of the Crested Ibis are close to the north and south in this study. The principal reason is that the Crested Ibis need tall trees for egg-laying and nesting during the breeding season [18], and there are many fruit groves and economic forests in the north and south of the Yang-xian Reserve in Shaanxi Province providing a large amount of forest land for roosting.

There are numerous factors influencing Crested Ibis’ habitat selection. The analysis shows that NDVI makes the largest contribution to the habitat assessment model and largely reflects habitat quality. We believe the NDVI helps interpret the impact of the habitat of the Crested Ibis, and it also validates the fact that vegetation changes have an important effect on the survival of the Crested Ibis [10,14]. It can also explain the conflicts with the findings of Zheng, L.’s research suggesting that altitude is the main factor affecting the habitat of the Crested Ibis, without considering the vegetation [22]. Besides the vegetation, the altitude and distance from rivers also influence the Crested Ibis’ habitat selection. The high-altitude areas are consistent with the Crested Ibis’ habit of choosing nocturnal roosting [6], and rivers can provide more fish, shrimps, and crabs [9,12]. Taking Luoshan County as an example, there are large areas (including many protected areas) suitable for the Crested Ibis to survive, especially in the southern areas. Considering the fact that the Crested Ibis usually reuse the same breeding site without frequent human disturbance [13], we advocate the protection of paddy fields and reservoirs for local farmers where the Crested Ibis once stayed, encourage active water-storage in winter, and prohibit the extensive use of pesticides for maintaining a stable ecological environment.

Human behavior also has a considerable impact on the habit selection of the Crested Ibis [11,12,43], and we believe this kind of impact should be evaluated more comprehensively. On the one hand, some artificial features could provide adequate food for the Crested Ibis; on the other hand, human activities might disturb the life of the Crested Ibis [22]. There are developed paddy fields and ponds along the roads, which are conducive to the Crested Ibis’ survival. However, human activities probably disturb the life of the Crested Ibis, and the continued expansion of the road network could also threaten the survival of the Crested Ibis [44]. Thus, revealing the relationship between Crested Ibis survival and human activities needs further study, and it might depend on specific cases.

There is a potential problem that might affect the accuracy of the analysis, which is the uneven data distribution caused by the missing trajectory data [45,46,47]. To deal with the problem, we built an LSTM module to supplement missing data and ensure its integrity in this study. Compared to the Skip-gram model [45], the LSTM module is more suitable for predicting the trajectories of individual Crested Ibis. The LSTM model is able to preserve information during long periods for learning-order dependence in sequence prediction problems [48]. This result also validates Wijeyakulasuriya, D. A.’s study, which adopted LSTM to predict the trajectory of the Larus fuscus [47]. Although the LSTM module can supplement the trajectory data, this supplementary process also generates errors for habitat prediction. Thus, in the future study, we will evaluate the prediction and obtain more Crested Ibis trajectory-data for broader analysis.

## 5. Conclusions

Based on the spatial and temporal trajectory-data and multi-source geographic and environmental data of the Crested Ibis, this paper uses the clustering method suitable for Crested Ibis to determine its habitat. Moreover, we analyze the biological patterns and behavioral patterns of the Crested Ibis, including the characteristics of night roosting points, the types of roosting vegetation, and the distance analysis between night roosting points and foraging points. Since the trajectories of the Crested Ibis are missing, to different degrees, an LSTM network was constructed to supplement the missing trajectory data. Additionally, this paper predicts the habitat distribution of the Crested Ibis in Luoshan County using a random forest method adjusted for its suitability, and discusses the potential habitat conditions, the impact of human activities, and the current habitat status and conservation status of the surrounding environment.

This paper provides a complete analysis tool and process for the analysis of spatial and temporal trajectory-data of the Crested Ibis, and provides methods and suggestions for experts to make future decisions.

## Figures and Tables

**Table 1:** Crested Ibis Data Sheet. ID is the number of each individual; date is the time range of each individual’s data recording; numbers of trajectory is the number of trajectory points for each individual.

ID	Date	Location	Numbers of Trajectory
4B04A0	16 July 2014–30 December 2021	Luoshan County, Henan Province	6541
4B03DB	16 July 2014–30 December 2021	Luoshan County, Henan Province	1878
CAFL004	24 July 2015–30 December 2021	Hantai District, Chenggu County, Yang County in Shaanxi Province	18,600
8013	24 July 2015–30 December 2021	Yang County in Shaanxi Province	220
8014	24 July 2015–30 December 2021	Yang County in Shaanxi Province	2769
8022	24 July 2015–30 December 2021	Chenggu County, Yang County in Shaanxi Province	947
8026	24 July 2015–30 December 2021	Chenggu County, Yang County in Shaanxi Province	870

**Table 2:** Environmental data sets used in the analyses, and their sources and resolution. Resources can be downloaded from the website in the citation following the data name.

Name	Sources	Resolution(m)
Google Maps	Satellite LANDSAT-8, QuickBird, etc.	0.5 m
DEM (digital elevation model) [32]	Geospatial Data Cloud	30 m
GlobeLand30 land cover data [33]	GlobeLand30	30 m
NDVI (normalized difference vegetation index) [32]	Geospatial Data Cloud	250 m
River vector data [34]	National Catalogue Service for Geographic Information	/
Road vector data [35]	OpenStreetMap database	/

**Table 3:** Selection rules of study periods for location points.

Location Points	March–October	November–February
Night roost points	21:00–5:00	20:00–6:00
Foraging points	8:00–16:00	9:00–16:00
Outing points	5:00–8:00 16:00–21:00	6:00–7:00 16:00–20:00

**Table 4:** Evaluation indicators. *R*^2^ is the degree of model fit; *RMSE* is the root-mean-square error; *MAE* is the mean absolute error; explained variance is the degree of explanation of the model.

Precision Evaluation Indicators	*R* ^2^	*RMSE*	*MAE*	Explained_Variance
Value	84.9%	21.0	12.6	85.7%

## Data Availability

The trajectory data are available upon request from the corresponding author. Additional environmental data can be obtained from the data sources in Table 2.

## Supplementary Materials

The following supporting information can be downloaded at https://www.mdpi.com/article/10.3390/ani13020237/s1, Figure S1: Loss graph. The horizontal coordinate is the epoch in the training process, and the vertical coordinate is the output value of the loss function; Figure S2: Clustering schematic—DBSCAN (left) Kmeans (right); Figure S3: optimal parameters: silhouette score (left) signal-to-noise ratio (right). The horizontal coordinates are Minpts, ranging from 1 to 20; the vertical coordinates are the silhouette score and the noise ratio. The silhouette score was close to 1 and the noise ratio was relatively small when Minpts was 3; Figure S4: Error bar graph. The horizontal coordinate is the group ID, and the vertical coordinate is the MSE score. Error bars represent the standard deviation of scores for each group. Since the error of group 10 is too large, it is not shown in the figure; Table S1: Random Forest Parameter Tuning. *n_estimators* is the number of subtrees built for the whole model; *min_samples_split* is the minimum number of samples required for node division; *min_samples_leaf* is the minimum number of leaf nodes; *max_features* is the maximum number of features selected; *max_depth* is the maximum depth of the tree; *bootstrap* is whether the sampling method selects bootstrap; Table S2: Random forest optimal parameters.
