# Supplementary material for: Spatial- and Temporal-Trajectory Analysis of the Crested Ibis (Nipponia nippon) by Fusing Multiple Sources of Data
Source: Animals (Basel). 2023 Jan 9;13(2):237. doi: 10.3390/ani13020237 (PMC9854527; doi:10.3390/ani13020237)
Supplement: Supplementary file 1 [file animals-13-00237-s001.zip › animals-2080956-supplementary.pdf]

animals

MDPI

animals-2080956 Supplementary Meterials

1. LSTM (Long Short-Term Memory)

Four fully connected layers were set in LSTM, and the number of hidden units was the same, set to 128. *Tanh* is centered on 0 compared to sigmoid, which makes its convergence faster than *sig* and can reduce the number of iterations, and the amount of data in this experiment is large.Therefore, considering the time complexity, *tanh* was chosen as the activation function.

$$\text{tanh}: f(x) = \frac{e^x - e^{-x}}{e^x + e^{-x}} \tag{1}$$

The Dropout layer was designed to prevent overfitting of the neural network because of the overfitting phenomenon during the neural network training. The Dropout continued to delete neurons and update parameters until the 100th batch was done. When the state of the neurons remained unchanged, the entire Dropout layer finished its function. The whole Dropout layer was finished.

The reason for adding the activation layer was to introduce a non-linear factor. The activation function allowed the latitude and longitude sequence data to be better fitted to the model, preventing the output of each layer from being a linear function of the previous input. ReLU caused the output of a portion of the neurons to be zero and caused sparsity in the grid, reduced parameter interdependencies, mitigated the occurrence of overfitting problems, and kept the gradient constant for gradients greater than one, without causing the gradient to disappear.

The latitude and longitude series data $[[lon_{1'},lat_{1'}],[lon_{2'},lat_{2'}],[lon_{3'},lat_{3'}]\ldots[lon'_{n},lat'_{n}]]$ were weighted, added together, and then calculated using the activation function *ReLU*.

$$f(x) = max(0, x) \tag{2}$$

The LSTM-model design used for the supplementary data was trained for 100 epochs, using MSE as the loss function, with the loss converging to stability after approximately 80 epochs (Figure S1). The haversine distance error for the test set was calculated to be 50 m to 194 m, and the error was mostly concentrated between 63 m and 100 m, with a mean error of 85 m, which was within the ideal range. The standard deviation was 28 m, and the fluctuation in error was relatively good.

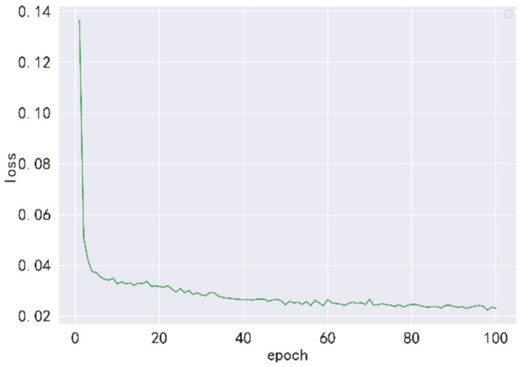

Figure S1. Loss graph. The horizontal coordinate is the epoch in the training process, and the vertical coordinate is the output value of the loss function.

2. DBSAN

Compared to the K-means method, the DBSCAN [38] method has a good adaptability to non-convex clusters, its clustering benefits are unaffected by noise, and the clustering results achieve global optimality [39].

K-means divides the sample set into K cluster,s according to the distance between samples. The points in the cluster are made as close as possible, and the distance between the clusters is made as large as possible [40]. Let the clusters be divided into $[C_1, C_2,...C_k]$, then the goal is to minimize the squared error E:

$$E = \sum_{i=1}^{k} \sum_{x \in C_i} \left|\left|x - \mu_i\right|\right|^2 \tag{3}$$

μ_i is the mean vector of clusters C_i:

$$\mu_i = \frac{1}{|C_i|} \sum_{x \in C_i} x \tag{4}$$

Compared with K-means, the DBSCAN algorithm selects night roosting points and marks noise. The neighborhood of the sample point is recorded as *Eps*, the minimum number of samples of the cluster class is recorded as *MinPts*, and the data set is recorded as *D* [38].

Define a point in the neighborhood *Eps* of a point *p* as *q*, and the point *q* constitutes a set $N_{Eps}\ (p)$.

$$NEps\ (p) = \{q \in D | dist(p, q) < Eps\} \tag{5}$$

Define density reachable, calculated as follows:

$$q \in N_{Eps}\ (q) \tag{6}$$

$$|N_{Eps}(q)| > MinPts \tag{7}$$

Define $[C_1, C_2,...C_k]$ as the clusters in dataset *D*, parameters Eps_i and $MinPts_i$, $i=1,...,k$, and then we define the noise as the point set *D* in the database that does not belong to any cluster C_i. The noise point is calculated as follows:

$$noise=\{p \in D | \forall i: p \notin C_i\} \tag{8}$$

Take ibis 4B04A0 as an example (Figure S2). It is difficult for Kmeans to find real clusters, as two points from different clusters may be closer than two points in the same cluster. Additionally, DBSCAN can efficiently identify noisy points, while Kmeans divides the noisy points into clusters. In this paper, the clustering algorithm of DBSCAN, which is suitable for the spatial and temporal track-data of the crested ibis, was used to identify its habitat and noise points that may be disturbed by human factors after tuning it according to the characteristics of the crested ibis. Based on the algorithm's results, the behavioral patterns and biological patterns of the crested ibis were initially analyzed by fusing multiple environmental data sources.

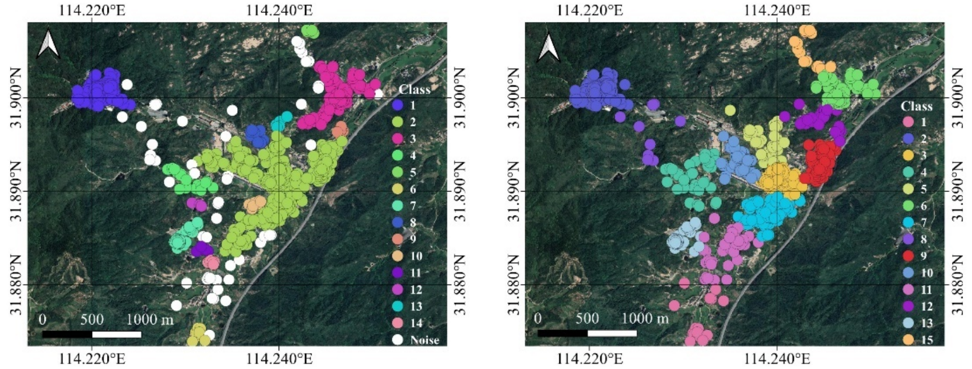

Figure S2. Clustering schematic—DBSCAN (left) Kmeans (right).

To evaluate the suitability of the clustering parameters for the ibis' spatial and temporal data, the silhouette coefficient proposed by Peter J. Rousseeuw (1986) was used [41]. The closer the silhouette coefficient is to 1, the better the suitability of the clustering parameters for the spatial and temporal data of the crested ibis. For the selection of the night roosting points, a neighborhood radius of 1 km was chosen by referring to the definition in the International Crested Ibis Conservation Workshop.

Where the silhouette coefficients were defined for each sample, the distance metric adopted Euclidean distances, and consisted of two components.

a: the average distance of the sample from all other points in the same class.

b: the average distance of the sample from all other points of the next nearest class.

Then for each sample, the silhouette coefficient, s

$$s = \frac{b - a}{max(a, b)} \tag{9}$$

The silhouette coefficients for a set of sample points were obtained by taking the mean of the silhouette coefficients for each sample. The signal-to-noise ratio was the ratio of noise points to the total number of samples. Finally, the values of the silhouette coefficients and the S/N ratio were combined to determine the *Minpts* (minimum number of samples in the neighborhood).

The range of *Minpts* (minimum number of samples in the neighborhood) was set from 1 to 20. The silhouette score of the sample data set was close to 1 when *Minpts* was 3, and the noise ratio was relatively small, so the *Minpts* parameter was chosen to be 3 (Figure S3).

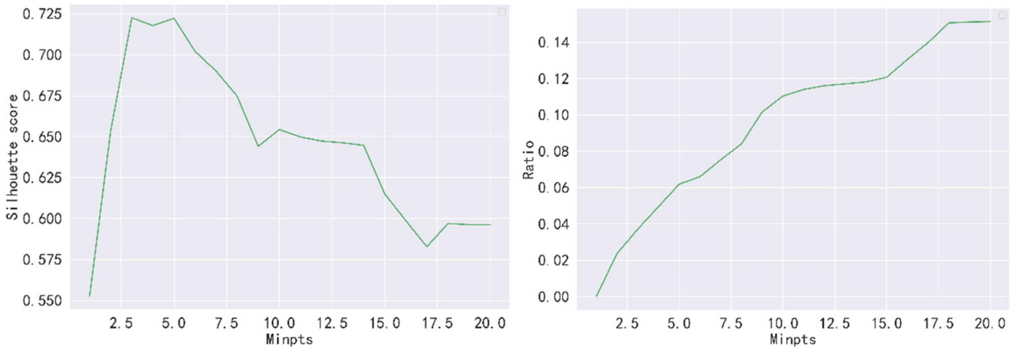

Figure S3. optimal parameters: silhouette score (left) signal-to-noise ratio (right). The horizontal coordinates are *Minpts*, ranging from 1 to 20; the vertical coordinates are the silhouette score and the noise ratio. The silhouette score was close to 1 and the noise ratio was relatively small when *Minpts* was 3.

3. Random Forest

Training samples were obtained by repeated multiple sampling with put-back using the bagging method, and this sampling result was used as the training set for generating the decision tree. Using the four input feature-variables, *n* feature variables ($n < 4$) were randomly selected for each node in the tree. The best splitting point for the decision tree was determined with the *n* feature variables selected. Each decision tree (classifier) was grown to its maximum capacity but not pruned, and the results of all decision trees were finally averaged to construct a regression model.

To make the random forest model perform optimally on ibis data, achieve higher model effectiveness, achieve better fitness and fit, and improve the stability of the model predictions, parameter tuning was required. The parameters were tuned by choosing the random search and collapse method, and the number of *n_estimators* to build subtrees needed to be determined before using the mean to make predictions. A higher number of subtrees would allow for higher model-effectiveness and the best possible prediction

stability within hardware performance limits. Another parameter to be adjusted was the end nodes of the decision tree, also known as leaf nodes *min_samples_leaf*. A smaller number of leaf nodes made capturing the noise in the training dataset easier. Further parameters include the maximum depth of the tree, *max_depth*, the minimum number of samples required for node splitting, *min_samples_split*, the maximum number of features required for node splitting, *max_features,* and whether the sampling had a put-back bootstrap parameter. These parameters were arranged and combined to obtain ten grouped representations, which were fitted 30 times, and the best combination of parameters was found using the MSE scores.

In this paper, random sampling was used in the random forest method to ensure that the training samples were different for each tree, maintaining the variability of the training results for each tree and ensuring that the results were meaningful when the regression was calculated. Random sampling is a put-back sampling, and since the training samples of each tree are different, if there is no intersection between trees, each tree will be biased. We need to ensure that the trees are completely fair to each other, and then use a mean-taking decision in the regression calculation to produce the results to prevent overfitting. Multiple training sets were generated by bootstrap sampling for different CART tree models, and then the output of each tree was averaged by bagging to reduce the variance of the output target statistic. After tuning all CART trees with parameters and taking the mean of the calculated results to obtain the trained regression model, the impact factors' evaluation was normalized to obtain the habitat-rank evaluation results.

We fed multi-source data into the regression model and normalized the results. Finally, we ranked potential habitats from 1 to 10. The min-max-scaling-normalization method, X_{scaled},was used here.

$$X_{std} = \frac{(x_i - min[x_1 \ldots x_i])}{max[x_1 \ldots x_i] - min[x_1 \ldots x_i]} \quad (10)$$

$$X_{scaled} = X_{std} * (max[x_1 \ldots x_i] - min[x_1 \ldots x_i]) + min[x_1 \ldots x_i] \quad (11)$$

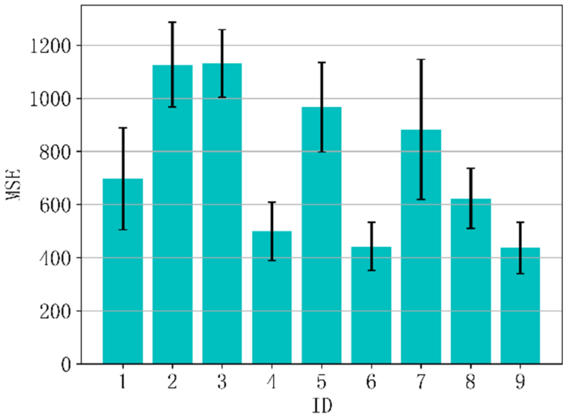

Figure S4. Error bar graph. The horizontal coordinate is the group ID, and the vertical coordinate is the MSE score. Error bars represent the standard deviation of scores for each group. Since the error of group 10 is too large, it is not shown in the figure.

The combination of grouping ID of 6 (refer to Table S1) has the smallest MSE score and the smallest error bar, so the parameters of grouping 6 perform optimally (Figure S4). The number of subtrees built for the whole model is 1200; the minimum number of leaf nodes is 1; the maximum depth of the tree is 70; the minimum number of samples required for node division is 5; the maximum number of features is automatically selected, and the sampling method with put-back is chosen (Table S2).

Table S1. Random Forest Parameter Tuning. *n_estimators* is the number of subtrees built for the whole model; *min_samples_split* is the minimum number of samples required for node division; *min_samples_leaf* is the minimum number of leaf nodes; *max_features* is the maximum number of features selected; *max_depth* is the maximum depth of the tree; *bootstrap* is whether the sampling method selects bootstrap.

ID	n_estimators	min_samples_split	min_samples_leaf	max_features	max_depth	bootstrap
1	1000	10	1	sqrt	70	FALSE
2	1600	10	4	auto	90	FALSE
3	200	10	2	sqrt	50	TRUE
4	1000	2	2	auto	80	TRUE
5	200	10	2	auto	70	TRUE
6	1200	5	1	auto	70	TRUE
7	600	10	4	sqrt	100	FALSE
8	600	2	2	auto	70	FALSE
9	1800	5	1	auto	100	TRUE
10	2000	2	1	auto	20	FALSE

Table S2. Random forest optimal parameters.

n_estimators	min_samples_leaf	max_depth	min_samples_split	max_features	max_features
600	2	70	2	auto	auto
